# Longitudinal Effects of Stress and Cognitive Fusion in Anxiety and Depressive Symptoms of Family Caregivers

**DOI:** 10.1093/geroni/igab046.1076

**Published:** 2021-12-17

**Authors:** Samara Barrera-Caballero, Rosa Romero Moreno Carlos Vara-García, Javier Olazarán, María del Sequeros Pedroso Chaparro, Lucía Jiménez-Gonzalo, José Adrián Fernandes-Pires, Andrés Losada-Baltar

**Affiliations:** 1 Rey Juan Carlos University, Alcorcón, Madrid, Madrid, Spain; 2 Universidad Rey Juan Carlos, Madrid, Madrid, Spain; 3 HGU Gregorio Marañón,, Madrid, Madrid, Spain; 4 Universidad Autónoma de Madrid, Madrid, Madrid, Spain; 5 Rey Juan Carlos University, Alcorcón, Madrid, Spain

## Abstract

Dementia caregiving has been commonly associated with negative psychological consequences in caregivers. Cognitive fusion, that is, the tendency for been overly influenced by cognition, has been linked to psychological distress in caregivers in cross-sectional studies. Female caregivers and those who are exposed to more stressors such as behavioral and psychological symptoms of dementia report higher levels of distress. However, longitudinal analysis of predictors of caregivers levels of distress are sparse, with no available study analyzing the longitudinal effect of cognitive fusion. The aim of this study is to analyze the longitudinal effect of cognitive fusion in depressive and anxiety symptoms of family dementia caregivers, after controlling for other relevant variables. Face to face interviews were conducted each year through a two-year period (three assessments) with 143 caregivers. Linear mixed models analysis were used to analyze the associations between time-varying values for cognitive fusion, frequency and reaction to care-recipient behavioral problems and depressive and anxiety symptoms, after controlling for caregivers’ age and gender, daily hours and time caring, care-recipient functional capacity and caregivers’ transitions (cessation of caregiving). Results suggest that increases in cognitive fusion and in reaction to behavioral problems, being a female caregiver and being younger, significantly predicted increases in anxiety symptoms over time. Also, increases in cognitive fusion and in reaction to behavioral problems, decreases in care-recipient’s functional capacity and ending of the caregiving role significantly predicted increases in depressive symptoms. Psychological strategies aimed at reducing cognitive fusion and stress levels may be especially helpful for reducing caregivers’ distress.

